# Prescribing Empiric Antibiotics for Febrile Neutropenia: Compliance with Institutional Febrile Neutropenia Guidelines

**DOI:** 10.3390/pharmacy6030083

**Published:** 2018-08-10

**Authors:** Doaa Naeem, Majed A. Alshamrani, Mohammed A. Aseeri, Mansoor A. Khan

**Affiliations:** Pharmaceutical Care Department, Ministry of National Guard Health Affairs, King Saud bin Abdul Aziz University for Health Sciences, King Abdul Aziz Medical City, Jeddah 11481, Saudi Arabia; Naeemdoaa@gmail.com (D.N.); ShamraniMA01@ngha.med.sa (M.A.A.); KhanMA1@ngha.med.sa (M.A.K.)

**Keywords:** empiric, antibiotics, febrile, neutropenia, compliance

## Abstract

**Background:** Febrile neutropenia (FN) is an oncologic emergency which should be treated immediately with empiric antibiotics. Different institutions observe different antibiograms and use different FN management guidelines. Our center implemented FN management guidelines for adult cancer patients in 2009. Hence, we decided to assess compliance with FN management guidelines and to describe the pattern of bacterial infections. **Method:** We conducted a cross-sectional study on all adult cancer patients admitted with FN. Data were collected from electronic medical records between January and December 2014. **Results:** One hundred FN episodes met the study inclusion criteria. The mean age of the patients was 41 ± 17 years; 52% (52 patients) were women. The most common diagnosis was lymphoma (33%). In terms of compliance to institutional FN guidelines, 55% of patients received guideline non-compliant treatment. The most common non-compliant treatment was incorrect amikacin dosing in 31% of patients, followed by incorrect vancomycin dosing in 20%, incorrect piperacillin/tazobactam dosing in 19%, inappropriate use of carbapenems in 18%, and non-compliant vancomycin use in 12% of patients. Bacterial isolates were only observed in 19% of the FN episodes. Among these 19 episodes of FN, Gram-negative pathogens were predominant and were identified in 74% of the episodes, followed by Gram-positive pathogens in 16% and polymicrobial pathogens in 10%. The mean time to defervescence was 2.21 ± 2 days. **Conclusion:** Our study concluded that there was a high percentage of non-compliance with our institutional FN management guidelines. We recommend following appropriate empiric antibiotic doses and indications as per institutional guidelines.

## 1. Introduction

Patients receiving chemotherapy frequently developed periods of neutropenia that might be complicated by fever. This fever may be the only alarming indicator of an infectious process happening due to the impaired signs and symptoms of inflammation. During neutropenic phase, health care providers must be extremely cautious about the risk of infections, diagnostic tools and the empirical antimicrobial choices to adequately cover the patient throughout the neutropenic period [1,2].

Fever is defined as a single oral temperature measurement ≥38.3 °C (101 °F) or a temperature ≥38.0 °C (100.4 °F) persistent over a 1-h period. While, neutropenia is defined as an Absolute Neutrophil Count [ANC] <500 cells/mm^3^, or an ANC anticipated decrease to <500 cells/mm^3^ in the next 48 h [1,2].

Fever occurs frequently during chemotherapy-induced neutropenia. Patients receiving chemotherapy for one or more cycles have developed febrile neutropenia in >80% of those with patients diagnosed with hematologic malignancies and 10–50% of patients with solid tumors [3]. Most patients with FN will have an unidentified focus of infection. The chance of clinically documented infections seen only in 20–30% of febrile episodes, and the most common sites of infection involve the intestine, lung, and skin [1,2].

While individuals with cancer experience many side effects from treatment, infection is the most common complication. Neutropenia is a major risk factor for the development of several types of infections. Although bacteremia is documented in only 15% to 20% of neutropenic patients, it represents a major cause of death in febrile neutropenic patients. The risk of infection is significantly linked to the grade and duration of neutropenia [4].

Defervescence is defined as a reduction in orally measured body temperature to <38 °C that is sustained over at least 48 h [5]. Many studies have suggested that the time to defervescence in febrile neutropenic patients is 2–7 days with a median of 5 days [6].

In 2009, King Abdulaziz Medical City-Western Region (KAMC-WR) created and implemented febrile neutropenia (FN) management guidelines, and a clinical pathway for adult patients to reduce FN patient morbidity and mortality. These guidelines were adapted from the Infectious Diseases Society of America (IDSA) guidelines for the use of antimicrobial agents in cancer patients with febrile neutropenia which were firstly published in 2002 and were later changed according to the updated IDSA 2010 clinical practice guidelines [7].

The purpose of the guidelines was to provide evidence-based treatment and optimal care for FN patients and to reduce the emergence of antibacterial resistance in the KAMC-WR hematology/oncology population. Our institutional antibiogram in 2012 showed 30–40% Gram-negative resistance to β-lactams. A rapid increase in Gram-negative pathogen resistance to the standard antibiotics used as empirical therapy for FN drove the assessment of compliance with our empiric antibiotic therapy recommendations for FN outlined in the KAMC-WR FN guidelines [7].

According to our KAMC-WR adult FN clinical pathway, antibiotics should be initiated as soon as possible following blood cultures; either dual therapy (piperacillin/tazobactam and amikacin) for 3 days with a reevaluation after 3 days or triple therapy (piperacillin/tazobactam, amikacin and vancomycin). In the case of penicillin allergy, ciprofloxacin, amikacin and vancomycin can be initiated [7] (See Table 1).

Dual therapy rather than monotherapy is suggested because of the 15–30% Gram-negative resistance to β-lactams at our institutions. The use of ceftazidime is discouraged because it is associated with breakthrough Gram-negative resistant infection. Imipenem/meropenem should be avoided as initial therapy because of the risk of *Clostridium difficile* and *Stenotrophomonas* infection, unless the patient is hemodynamically unstable or the patient has a history of Extended Spectrum Beta-Lactamase (ESBL).

Different institutions have different antibiograms, leading to different microbiological infection patterns in FN patients. We performed an extensive literature search and found no published studies addressing compliance with empirical antibiotics guidelines and bacterial infection patterns in FN patients in Saudi Arabia, prompting us to assess compliance levels during FN patient treatment.

### 1.1. Primary Objective

The objective of the study was to assess compliance with institutional guidelines during FN management.

### 1.2. Secondary Objectives

- To describe the pattern of bacterial infections among patients with FN at our institution.
- Determine the microbiological sensitivity of the bacterial isolates.
- Identify time to defervescence in FN patients.

## 2. Method

### 2.1. Inclusion Criteria

All adult cancer patients (age > 14 years) hospitalized with febrile neutropenia between January 2013 and December 2014 were included. 

### 2.2. Exclusion Criteria:

- Age < 14 years
- FN but were not enrolled in the FN clinical pathway at the time of admission

### 2.3. Study Design

In this retrospective cross-sectional study, data were collected from the FN clinical pathway and electronic medical records for all febrile neutropenic episodes in cancer patients admitted during the 1-year period from January 2014 to December 2014 at KAMC-WR.

This study was conducted at King Abdulaziz Medical City, a 500-bed tertiary care teaching medical center treating the largest number of adult and pediatric cancer patients in the western region.

Patients with fever and neutropenia were hospitalized mainly in the Emergency Room (ER) or oncology/hematology wards. Our retrospective study included patients who had begun the FN clinical pathway and received empirical antibiotics. An empirical therapy for febrile neutropenia included dual therapy with piperacillin/tazobactam and amikacin or triple therapy with piperacillin/tazobactam, amikacin and vancomycin as per our institutional FN guidelines. 

KAMC-WR empirical therapy guidelines were considered complied with if the below empirical antibiotics and doses were used:

- Piperacillin/tazobactam 4.5 gm IV q6hr + amikacin 15 mg/kg Q24h as per IBW or in obese patients as per adjusted BW (15–20 mg/kg/day was allowed and considered in compliance with the FN pathway)
- Piperacillin/tazobactam 4.5 gm IV q6hr + amikacin 15 mg/kg Q24h + vancomycin 15 mg/kg IV q12h if there is an indication for adding vancomycin
- Ciprofloxacin 400 mg IV q8hr + amikacin 15 mg/kg Q24h + vancomycin 15 mg/kg IV q12h (in the patient with penicillin allergy)
- Carbapenem (meropenem 1000 mg q8hr or imipenem 500 mg q6hr) in the case of ESBL
- All of the empirical antibiotics require renal dose adjustment (For the appropriate renal adjustment, see Appendix A)
- Compliance with empirical vancomycin use including one or more of the indications mentioned in Table 1

KAMC-WR empirical therapy guidelines were not considered complied with if the below empirical antibiotics/dosages are used:

- Piperacillin/tazobactam monotherapy
- Piperacillin/tazobactam + amikacin (one or both at the wrong dose)
- Piperacillin/tazobactam + amikacin + vancomycin (any used at the wrong dose or with no indication for vancomycin)
- Carbapenem (with no ESBL history)
- Piperacillin/tazobactam + amikacin (in patient with penicillin allergy)
- Dosages of empirical antibiotics were not adjusted to consider renal impairment

### 2.4. Sample Size

All of the patients hospitalized with FN that began the FN pathway when admitted to our oncology center at KAMC-WR during the period of January 2014 until December 2014 and that fulfilled the inclusion criteria were included. 

## 3. Data Management and Analysis Plan

Descriptive statistics (percentages and proportions) were used to assess compliance with institutional guidelines for FN management regarding empirical antibiotic therapy and the pattern of bacterial infection.

## 4. Results

One hundred febrile neutropenic episodes were identified that met the study inclusion criteria. The mean age of the study population was 41 ± 17 years; 52% (52 episodes) were women, and 48% (48 episodes) were men. Lymphoma was the most common diagnosis in 33 patients (33%), followed by acute leukemia in 34 patients (34%) and breast cancer in four patients (4%); 29 patients (29%) had other tumors diagnose. The baseline characteristics of the study patients are described in Table 2. Compliance with the institutional FN management guidelines regarding the use of empirical antibiotic management in FN patients was 45% compared with 55% of patients that received non-compliant treatment. The most common non-compliance was incorrect amikacin dosing 31%, followed by incorrect vancomycin dosing 20%. Furthermore, incorrect piperacillin/tazobactam dosing was observed in 19% of cases, inappropriate use of carbapenem in 18% of cases, and the inappropriate use of vancomycin in 12% of cases. Bacterial isolates were only found in 19% of episodes and were predominantly Gram-negative pathogens (16% of episodes), whereas Gram-positive bacterial isolates were observed in 4% of episodes. Among the 19 cases of FN in which bacterial isolates were identified, Gram-negative pathogens were identified in 74% (16 episodes), followed by Gram-positive bacterial isolates in 16% (3 episodes) and polymicrobial pathogens in 10% of episodes. Among the Gram-negative bacteria, *Escherichia coli* was the most common pathogen with six isolates: four of the six isolates were ESBL, and two isolates were sensitive to penicillin. Three isolates of *Klebsiella pneumonia* were identified, one of which was ESBL, and the remaining two were sensitive to penicillin. Three isolates of *Pseudomonas aeruginosa* were observed, and all of them were sensitive to ceftazidime and piperacillin/tazobactam. Three isolates of *Salmonella* were sensitive to ceftriaxone, and two of the three isolates were resistant to ciprofloxacin. Two isolates of *Acinetobacter baumannii* were identified, one of which was resistant to imipenem, and both isolates were sensitive to colistin. Five isolates of Gram-positive bacteria were identified, specifically two isolates of *Enterococcus* species sensitive to ampicillin, one isolate of *Micrococcus luteus* that was sensitive to piperacillin/tazobactam, coagulase-negative *Staphylococcus aureus* sensitive to vancomycin, and methicillin-sensitive *Staphylococcus aureus*. The mean time to defervescence in FN patients was 2.21 ± 2 days. 

## 5. Discussion

FN is a medical emergency, and patients suspected to have an FN infection should be treated with empiric, broad-spectrum antibiotics immediately after collecting a culture sample. Patients are stratified based on the risk level to low risk and high risk during the FN phase directing all recommendations with regard to evaluation, therapy selection, treatment settings, and prophylaxis [8]. “High risk patients necessitate hospital admission for empirical intravenous antimicrobials; monotherapy with an antipseudomonal β-lactam agent, piperacillin-tazobactam, cefepime, meropenem or imipenem-cilastatin is recommended as per the updated IDSA clinical practice guidelines for the use of antimicrobial agents in neutropenic patients with cancer. Other agents such as aminoglycosides or fluoroquinolones can be added empirically to target a suspected source (e.g., pneumonia or urinary tract infection), for hemodynamic instability (sepsis and hypotension) or if antimicrobial resistance (e.g., ESBL) is suspected or confirmed [1]. Medications active against Gram-positive cocci such as vancomycin or other agents is not recommended as a standard component of the initial antimicrobial regimen to treat FN. Those broad-spectrum antibiotics must be used only in particular situation like suspected catheter-related infection, skin or soft tissue infections, hemodynamic instability or pneumonia documented radiographically [1,2].

During the past 4 decades, considerable fluctuation has occurred in the epidemiologic spectrum of bloodstream isolates cultured from FN patients. Early in the development of cytotoxic chemotherapy in the 1960s and 1970s, Gram-negative pathogens were predominated [9]. Then, in the 1980s and 1990s, Gram-positive pathogens became more common due to the increased use of indwelling plastic venous catheters, which allow for colonization by and entry of Gram-positive skin flora [9]. Currently, coagulase-negative staphylococci are the predominant blood isolates at most centers; then enterobacteriaceae (e.g., *Enterobacter* species, *Escherichia coli* and *Klebsiella* species) and nonfermenting gram-negative rods (e.g., *Pseudomonas aeruginosa* and *Stenotrophomonas* species) are isolated less common [10,11,12].

We present data on FN management in adult cancer patients at a single oncology center in Saudi Arabia over one year. The study population primarily included cancer patients with solid tumors. Among the bacterial isolates, Gram-negative bacteria were most abundant. In contrast to findings at other centers that demonstrated a shift toward Gram-positive infection during the same period, *E. coli, P. aeruginosa* and *K. pneumonia* were the most common bloodstream isolates in our center. The identification of a specific gap between the institutional guidelines for FN management and current practice will facilitate updating currently available hospital guidelines. According to our research, we need to adjust the FN pathway depending on recent hospital antibiograms and microbial sensitivity, because our cancer center must monitor infection epidemiology among neutropenic cancer patients to guide empiric antibiotic treatment and decisions regarding antibiotic prophylaxis. We recommend providing clear instruction for dosing, especially for amikacin dosing, using ideal body weight and implementing education sessions for physicians and pharmacists regarding dose calculations. The limitation of our study is that it was retrospective study and conducted only in single cancer center.

## 6. Conclusions

Our retrospective study concludes that there is significant non-compliance with FN management guidelines regarding the use of empirical antibiotics. We recommend following the appropriate dose of empirical antibiotics, such as amikacin, vancomycin and piperacillin/tazobactam, and abiding by the appropriate indications for the use of carbapenems and vancomycin as per institutional guidelines.

## Figures and Tables

**Table 1:** The KAMC-WR clinical practice guidelines and clinical pathway for FN in adult patients.

Inpatient Empiric Antibiotics Regimen: Piperacillin/tazobactam 4.5 gm IV q6hr + amikacin 15 mg/kg IV q24hr as per Ideal Body Weight (IBW); If Morbidly Obese, Then Use the Adjusted Body Weight.
Indications for vancomycin addition to the empirical regimen for fever and neutropenia: 15 mg/kg IV q12h (trough 10–15 in non-responders):
1. Hemodynamic instability or other evidence of severe sepsis
2. Hemodynamic instability or other evidence of severe sepsis
3. Radiographically documented pneumonia
4. Positive blood culture for Gram-positive bacteria before final identification and susceptibility testing is available
5. Clinically suspected serious catheter-related infection (e.g., chills or rigors with infusion through a catheter and cellulitis around the catheter entry/exit site)
6. Colonization with methicillin-resistant *Staphylococcus aureus*, vancomycin-resistant enterococcus, or penicillin-resistant *Streptococcus pneumoniae*
7. Severe mucositis if fluoroquinolone prophylaxis has been given and ceftazidime is employed as the empirical therapy

**Table 2:** Baseline characteristics of study patients.

Characteristics	Number of Patients, N = 100
Age (mean ± SD)	44 ± 17 years
Sex, n (%)	Female, 52 (52%) Male, 48 (48%)
Diagnosis, n (%)	Lymphoma, 33 (33%) AML, 15 (15%) ALL, 19 (19%) Breast cancer, 4 (4%) Others, 29 (29%)

## Appendix A

**Table A1:** Appropriate antibiotic renal adjustment.

Piperacillin/tazobactam	**Dosage in Renal Failure** (**A**) All indications except nosocomial pneumonia (**1**) CrCl greater than 40 mL/min: No dose adjustment necessary (**2**) CrCl 20 to 40 mL/min: 2.25 g every 6 h (**3**) CrCl less than 20 mL/min: 2.25 g every 8 h (**B**) Nosocomial pneumonia (**1**) CrCl greater than 40 mL/min: No dose adjustment necessary (**2**) CrCl 20 to 40 mL/min: 3.375 g every 6 h (**3**) CrCl less than 20 mL/min: 2.25 g every 6 h					
Vancomycin	**CrCl (mL/min)**			**Vancomycin dose (mg/24)**		
	10			155		
	20			310		
	30			465		
	40			620		
	50			770		
	60			925		
	70			1080		
	80			1235		
	90			1390		
	100			1545		
Meropenem	CrCl > 50 mL/min: No dosage adjustment necessary. CrCl 26 to 50 mL/min: Administer the recommended dose based on indication every 12 h. CrCl 10 to 25 mL/min: Administer one-half of the recommended dose based on the indication every 12 h. CrCl < 10 mL/min: Administer one-half of the recommended dose based on the indication every 24 h.					
Imipenem and Cilastatin	**Body Weight (kg)**	**≥70**	**60**	**50**	**40**	**30**
	Total daily dose for normal renal function: 2 g/day					
	CrCl ≥ 71	500 mg q6h	500 mg q8h	250 mg q6h	250 mg q6h	250 mg q8h
	CrCl 41–70	500 mg q8h	250 mg q6h	250 mg q6h	250 mg q8h	125 mg q6h
	CrCl 21–40	250 mg q6h	250 mg q8h	250 mg q8h	250 mg q12h	125 mg q8h
	CrCl 6–20	250 mg q12h	250 mg q12h	250 mg q12h	250 mg q12h	125 mg q12h
Amikacin	GFR > 50 mL/min: No dosage adjustment necessary. GFR 10 to 50 mL/min: Administer every 24 to 72 h based on serum concentration. GFR < 10 mL/min: Administer every 48 to 72 h based on serum concentration.					

## Appendix C

**Table A2:** Microbiological sensitivity of the bacterial isolates.

	Specimen	Ampicillin	Oxacillin	Nitrofurantoin	Vancomycin	Linezolid	Amikacin	Cefazolin	Ceftriaxone	Ceftazidime	Cefepime	Ciprofloxacin	Gentamicin	Piperacillin/tazobactam	Septrin	Imipenem/Meropenem	Colistin	Tigecycline
*1. E. coli*	Blood									R	R	S	S	R	S	S		
*2. E. coli*	Sputum						S		R			S	R			S		
*3. E. coli*	Blood	R						R	R			R	S	R	S	S		
*4. E. coli*	Blood	R					S	R				R	R	R	R	S		
*5. E. coli*	Blood	R						S				S						
*6. E. coli*	Blood	S						S				S	S		S			
*7. Salmonella,* gp B	Blood	R					R	R	S			R	R		R			
*8. Salmonella,* gp B	Blood	R										S			R			
*9. Salmonella,* gp D	Blood	S							S			R						
*10. Acinetobacter baumannii*	Urine											S	S		S	S		
*11. Acinetobacter baumannii*	Sputum	R								R	R	S	S	R		R	S	S
*12. Klebsiella pneumonia*	Sputum									R	R	S	S	R		S		
*13. Klebsiella pneumonia*	Blood							S				S	S		S			
*14. Klebsiella pneumonia*	Blood	R						S				S	S					
*15. Micrococcus luteus*	Blood													S				
*16. Pseudomonas aeruginosa*	Urine									S		S	S	S				
*17. Pseudomonas aeruginosa*	Blood									S		S	S	S				
*18. Pseudomonas aeruginosa*	Blood									S		S	S	S				
*19. Staphylococcus coagulase negative*	Blood		R		S	S												
*20. Staphylococcus aureus*	Blood		S					S										
*21. E. faecalis*	Urine	S		S														
*22. E. casseliflavus*	Blood	S				S							S					

S: Sensitive. R: Resistance. The orange background color: gram negative isolates. The blue background: gram positive isolates.
